# Missed opportunities for family planning counselling among postpartum women in eleven counties in Kenya

**DOI:** 10.1186/s12889-022-12623-0

**Published:** 2022-02-08

**Authors:** Mary N. Thiongo, Peter B. Gichangi, Michael Waithaka, Amy Tsui, Linnea A. Zimmerman, Scott Radloff, Marleen Temmerman, Saifuddin Ahmed

**Affiliations:** 1 International Centre for Reproductive Health, Mombasa, Kenya; 2 Technical University of Mombasa, Mombasa, Kenya; 3 Department of Population, Family and Reproductive Health, Johns Hopkins Bloomberg school of Public Health, Baltimore, MD USA; 4 Aga Khan University, Nairobi, Kenya

**Keywords:** Contraception, Family Planning, Maternal health service integration, Missed opportunity, mCPR, Unmet need, Kenya

## Abstract

**Background:**

Mothers may access medical facilities for their babies and miss opportunities to access family planning (FP) services. This study was undertaken to describe missed opportunities for FP among women within the extended (0–11 months) postpartum period from counties participating in Performance Monitoring and Accountability 2020 (PMA2020) surveys.

**Design and setting:**

This study analysed cross-sectional household survey data from 11 counties in Kenya between 2014 and 2018. PMA2020 uses questions extracted from the Demographic and Health survey (DHS) and DHS definitions were used. Multivariable logistic regression was used for inferential statistics with *p*-value of < 0.05 considered to be significant.

**Participants:**

Women aged 15-49 years from the households visited.

**Primary outcome measure:**

Missed opportunity for family planning/contraceptives (FP/C) counselling.

**Results:**

Of the 34,832 women aged 15-49 years interviewed, 10.9% (3803) and 10.8% (3746) were in the period 0–11 months and 12–23 months postpartum respectively, of whom, 38.8 and 39.6% respectively had their previous pregnancy unintended. Overall, 50.4% of women 0-23 months postpartum had missed opportunities for FP/C counselling. Among women who had contact with health care at the facility, 39.2% of women 0-11 months and 44.7% of women 12-23 months had missed opportunities for FP/C counselling. Less than half of the women 0-11 months postpartum (46.5%) and 64.5% of women 12 – 23 months postpartum were using highly efficacious methods. About 27 and 18% of the women 0-11 months and 12 – 23 months postpartum respectively had unmet need for FP/C. Multivariable analysis showed that being low parity and being from the low wealth quintile significantly increased the odds of missed opportunities for FP/C counselling among women in the extended postpartum period, *p* < 0.05.

**Conclusions:**

A large proportion of women have missed opportunities for FP/C counselling within 2 years postpartum. Programs should address these missed opportunities.

**Supplementary Information:**

The online version contains supplementary material available at 10.1186/s12889-022-12623-0.

## Introduction

Universal access to reproductive health is essential to achieve the Sustainable Development Goals (SDGs) on health [1, 2]. The benefits of family planning, including reduction of maternal death, delaying motherhood, avoiding unintended pregnancies and abortions and reduction of mother to child transmission of HIV, accrue to women themselves, their offspring [3], and society [4–6]. Some of those benefits result from increasing the proportion of births that are optimally spaced [7, 8]. The recommended “birth-to-pregnancy interval” or “interpregnancy interval,” which is the duration between two consecutive pregnancies [7, 9], is considered to be at least 24 months. There is an increase in maternal, newborn, and child morbidity and in early childhood mortality rates associated with shorter intervals, particularly those less than 18 months [10–13]. Lack of access and use of family planning/contraceptives (FP/C) in the postpartum period often lead to short birth intervals. Provision of quality FP/C services in the postpartum period has the potential to reduce both maternal and childhood mortality and morbidity arising from unsafe abortions and inadequate spacing of births, respectively [14–17]. Postpartum FP/C is a proxy marker for integration and quality of FP services [17, 18].

While the clinical definition of the “postpartum” period is 42 days after birth, many programs are adopting the extended-postpartum to extend traditional postpartum period up to 1 year after birth [12, 14, 18]. Extended postpartum period is critical in preventing pregnancies and their associated complications. There are several safe and effective FP/C methods that women can use at various points after delivery, such as postpartum intrauterine devices, sterilization (male and female), condoms and emergency contraception, to optimize birth spacing and limiting [15, 18].

The postpartum period offers multiple opportunities for healthcare providers to assess, provide or promote FP/C. In 2014, about 61% of all women 15-49 years in Kenya delivered in a health facility, 51% of mothers made a postpartum visit and 79% of the children 12-23 months received basic vaccines [19] indicating substantial number of women of reproductive age had the potential to access to services in the postpartum period.

These maternal and newborn health services utilizations are opportunities for women to receive FP/C services during the extended postpartum period [17, 18, 20, 21]. A study on integration of FP into non-FP services [22] and postpartum FP with maternal, newborn and child health services [23] found that integration can reduce unmet need and increase uptake of FP/C services. The estimated prospective postpartum unmet for FP/C among postpartum women within 23 months post-delivery in Kenya is 57% [24]. There is need therefore to study FP/C use and its determinants among women of reproductive age in Kenya. Performance Monitoring and Accountability 2020 (PMA2020), designed to track performance of Family Planning 2020 (FP2020) commitments by different partners, generates FP/C data from households in 11 counties in Kenya. In this paper, we report missed opportunities for FP/C counselling among women within the extended (0–11 months) postpartum period and women 12-23 months post-delivery from a nationally representative counties participating in PMA2020 surveys.

## Materials and methods

### Data source

Nationally representative data collected via Performance Monitoring and Accountability 2020 (PMA2020) surveys in Kenya from 2014 to 2018 was used. PMA2020 conducts surveys every 6 months to 1 year, providing Family Planning 2020 (FP2020), governments, and other stakeholders’ frequent information on contraceptive use, demand, and supply that can inform policies and programs and identify areas for improvement. For each round of data collection, a new sample of households is chosen. The EAs were refreshed in 2016 and maintained till 2018. The PMA2020 household and female survey uses a two-stage cluster sample design with residential area (urban and rural) and county as strata to draw a probability sample of households and eligible females (age 15-49 years) across eleven counties in Kenya. In the first stage of sampling eleven of Kenya’s 47 counties were selected, using probability proportional to size procedures. Within the counties, clusters or enumeration areas (EAs) were selected proportional to size with urban/rural stratification. The sample of EAs was powered to generate national and urban/rural estimates of all woman modern contraceptive prevalence rates (mCPR) with less than 3% margin of error. A comprehensive description of the survey design is detailed elsewhere [25]. All study procedures were performed as per the research guidelines and regulations by the Kenyatta National Hospital and University of Nairobi Ethics review committee and National Council for Science, Technology and Innovation.

### Study population

We analysed a subsample of women aged 15–49 years who had a birth in the last 0 – 11 months, i.e., women in the extended postpartum period [12, 14, 18] and the results were compared to those for women 12–23 months post-delivery. Since pregnancy is possible within the first 6 months postpartum and some have experienced menses return within the period [26], the extended postpartum period was further divided into two sub periods: 0–5 months, 6–11 months in some analyses.

### Study variables

#### Dependent variables

The main outcome of interest was missed opportunity for FP/C counselling at the health facility or both at the facility and at the community. A missed opportunity for FP/C counselling at the facility refers to any contact with a health care worker at the facility by a woman in the postpartum period, which does not result in the person being counselled on FP/C. The overall missed opportunity for FP/C counselling refers to no counselling at all on FP/C either at the facility (for postpartum women who visited a facility in the last 12 months) and at the community (for postpartum women who visited a health facility in the last 12 months and were not counselled on FP/C as well as those who had not visited a health facility in the last 12 months and were not reached for FP/C counselling at the community by health care workers. A flow diagram representing the definition is shown in the supplemental material Fig. 1 and Fig. 2. Other variables that were assessed include demand for FP/C, FP/C use and unmet need for FP/C among postpartum women. Family planning/contraceptive use include the use of modern (Intrauterine devices (IUDs), hormone implants, male and female sterilizations, injectables, contraceptive pills, condoms, diaphragms, spermicidal agents and emergency contraception) as well as traditional (periodic abstinence, withdrawal and other folkloric methods) FP/C methods. Unmet need for FP/C was defined as the percentage of fertile, sexually active women ages 15–49 who were not using contraception and did not wish to become pregnant at all (unmet need for limiting) or within the next 2 years (unmet need for spacing)). Women were considered to have a demand for FP/C if they wanted to delay, space or limit childbearing.

#### Independent variables

Analyses in this study were stratified by woman’s age (15-19, 20-24, 25-29, 30-34, 35-39, 40-44, 45-49 years); wealth quintiles, based on the asset index included in the national survey datasets (lowest, lower, middle, higher and the highest wealth quintiles); marital status (never married, married or in union, widowed/divorced/separated); level of education (none, primary, technical/vocational, secondary or higher); area of residence (urban or rural) and parity (No children, 1 or 2 children, 3 or 4 children, 5 or more children). Other important covariates considered in this analysis include county of residence and round of data collection.

### Data analysis

Descriptive statistics including percentage, means, medians and standard deviation were computed. Means were compared using t-tests while Chi-square tests were used to measure the association between the factors and outcome variables. To study the covariates of missed opportunity for FP/C, bivariate and multivariable logistic regression analysis was applied using enter method to calculate the odds ratio (OR) and adjusted odds ratio (aOR) of missed opportunity for FP/C. The complex survey design and weights (individual sampling weights for women) were taken into account during the analysis. STATA 15.0 statistical software was used for all analyses (Stata Corporation, College Station, TX, USA). *P* < 0.05 was considered significant.

## Results

From 2014 to 2018, a total of 34,832 women aged 15-49 years were interviewed. The response rate was between 97.0 and 99.0% for all the rounds. Table 1 shows the sociodemographic characteristics of all women who participated in the PMA surveys as well as women 0-11 and 12-23 months post-delivery. Overall, the majority (58.4%) of the respondents were between the ages 15 and 29 years. About a third (31.9%) had never been married, 64.6% were from rural areas while 4.3% had no formal education. About a third of the respondents had one to two children (33.6%) and the majority were sexually active (58.0%). About a third of the respondents were from high (higher/highest) wealth quintile (37.9%) (Table 1). Out of the 34,832 women who had participated in the seven rounds of PMA2020 data collection 3803 (10.9%) and 3746 (10.8%) were 0 – 11 months and 12 – 23 months post-delivery respectively. Among women 0 – 11 months and 12 – 23 months post-delivery, the majority were between the ages 20 – 24 years, were married or in a union, were from rural residence, had primary level education, were from the lowest wealth quintile, had one or two previous births, were sexually active and were residing in Nairobi County.

**Table 1:** Sociodemographic characteristics of all women, women 0-11 and 12-23 months post-delivery

Characteristic	All women (***N*** = 34,832)			Women 0 –11 months postpartum (***N*** = 3803)			Women 11 –23 months postpartum (***N*** = 3746)		
	N	N*	%	N	N*	%	N	N*	%
**Age group**									
15 – 19	6973	6833	19.6	455	453	11.9	281	273	7.3
20 – 24	6599	6799	19.5	1267	1306	34.4	1139	1147	30.6
25 – 29	6496	6719	19.3	1058	1075	28.3	1099	1135	30.3
30 – 34	5159	5172	14.9	629	608	16.0	710	705	18.8
35 – 39	4034	3842	11.0	288	268	7.0	355	337	9.0
40 – 45	3209	3160	9.1	88	76	2.0	122	113	3.0
45 – 49	2362	2306	6.6	18	16	0.4	40	35	0.9
**Marital Status**									
Never Married	11,093	11,127	31.9	540	571	15.0	410	437	11.7
Married/In a union	21,123	21,104	60.6	3141	3119	82.0	3137	3105	82.9
Widowed/Divorced	2591	2570	7.4	120	109	2.9	197	203	5.4
Missing/No response	25	31	0.1	2	4	0.1	2	1	0.0
**Residence**									
Rural	22,138	22,507	64.6	2545	2513	66.1	2513	2499	66.7
Urban	12,694	12,325	35.4	1258	1290	33.9	1233	1247	33.3
**Education**									
No Education	1706	1481	4.3	290	233	6.1	282	232	6.2
Primary	16,928	16,700	47.9	1979	1941	51.0	1998	1959	52.3
Technical/Vocational	710	689	2.0	89	85	2.2	85	74	2.0
Secondary	10,973	11,226	32.2	1000	1042	27.4	952	1008	26.9
Higher	4509	4728	13.6	445	503	13.2	429	473	12.6
Missing/No response	6	8	0.0	0	0	0.0	0	0	0.0
**Wealth Quintile**									
Lowest	7820	7299	21.0	1074	984	25.9	1050	954	25.5
Lower	7549	7389	21.2	885	861	22.7	891	868	23.2
Middle	6991	6951	20.0	741	727	19.1	686	693	18.5
Higher	6337	6285	18.0	593	609	16.0	616	609	16.3
Highest	6135	6908	19.8	510	622	16.4	503	622	16.6
**Parity**									
None	9758	9691	27.8	N/A	N/A		N/A	N/A	
1 – 2	11,333	11,694	33.6	2104	2188	57.5	2005	2058	54.9
3 – 4	8009	7832	22.5	1021	992	26.1	1032	1030	27.5
5+	5732	5615	16.1	678	623	16.4	709	658	17.6
**Sexually active**									
No	14,346	14,616	42.0	1356	1407	37.0	800	834	22.3
Yes	20,486	20,216	58.0	2447	2396	63.0	2946	2912	77.7
**Ever given birth**									
No	9708	9632	27.7	N/A	N/A		N/A	N/A	
Yes	25,074	25,141	72.2	3803	3803	100.0	3746	3746	100.0
Missing/No response	50	59	0.2		0	0.0	0	0	0.0
**Data collection round**									
Jun/Jul 2014	3754	3729	10.7	461	454	11.9	476	458	12.2
Nov/Dec 2014	4329	4324	12.4	487	493	13.0	472	462	12.3
Jun/Jul 2015	4396	4385	12.6	528	541	14.2	449	458	12.2
Nov/Dec 2015	4921	4895	14.1	492	525	13.8	509	538	14.4
Nov/Dec 2016	5885	5916	17.0	645	627	16.5	647	642	17.1
Nov/Dec 2017	5876	5895	16.9	575	581	15.3	602	590	15.7
Nov/Dec 2018	5671	5688	16.3	615	583	15.3	591	599	16.0
**County**									
Bungoma	3954	3039	8.7	433	332	8.7	461	361	361
Kakamega	1513	3033	8.7	150	293	7.7	149	296	296
Kericho	3691	3955	11.4	411	437	11.5	405	431	431
Kiambu	3212	3166	9.1	267	295	7.8	254	269	269
Kilifi	4069	3721	10.7	522	501	13.2	517	479	479
Kitui	3634	3396	9.8	300	266	7.0	365	333	333
Nairobi	3258	4930	14.2	360	567	14.9	322	518	518
Nandi	3816	3283	9.4	370	321	8.5	348	292	292
Nyamira	3317	2459	7.1	327	244	6.4	291	220	220
Siaya	2959	2902	8.3	393	374	9.8	381	378	378
West Pokot	1409	949	2.7	270	173	4.6	253	168	168
	**Mean (S.D.)**		**Median**	**Mean (S.D.)**		**Median**	**Mean (S.D.)**		**Median**
Age of the participants	28.5 (9.2)		27.0	25.9 (5.8)		25.0	27.1 (6.1)		26.0
Number of live births	2.6 (2.3)		2.0	2.7 (1.9)		2.0	2.8 (2.0)		2.0
Age at first birth	20.0 (3.7)		19.6	20.1 (3.7)		19.6	20.1 (3.9)		19.6
Age at first sex	17.5 (3.1)		17.0	17.5 (2.9)		17.0	17.4 (3.1)		17.0
Age at first use of FP	23.2 (5.6)		22.0	22.2 (4.5)		21.0	22.7 (4.8)		22.0

*N* Unweighted sample size, N* Weighted sample size, % Weighted sample proportion, *S.D.* Standard deviation

Among women 0 – 11 months and 12 – 23 months post-delivery, 0.5 and 4.6% were pregnant respectively at the time of the survey (Table 2). Among women within 2 years post-delivery, 69.1% were sexually active at the time of the survey (Table 2). Of all the pregnancies at the time of the survey, 23.8 and 57.4% among women 0 – 11 and 12–23 months post-delivery respectively were unintended (wanted later or not at all). Among the sexually active women, 38.8 and 39.6% of those 0–11 months postpartum and 12–23 months postpartum respectively had a previous unintended pregnancy. About 2.1% of sexually active women within the 12-month postpartum period and 3.8% of sexually active women 12 – 23 months post-delivery expressed a desire to have another pregnancy within 2 years (Table 2).

**Table 2:** Pregnancy risk and fertility desire among the 24,968 women who had ever given birth before

	0 - 11 months (extended postpartum) (N = 3803)			12 - 23 month since last birth (N = 3746)		
	N	N*	%	N	N*	%
Currently pregnant	19	19	0.5	181	172	4.6
Current pregnancy unintended						
Wanted later	3	3	15.0	69	63	36.8
Not wanted at all	2	2	8.8	37	36	20.6
Sexually active^a^	2447	2414	63.0	2946	2916	77.7
Previous unintended pregnancies among sexually active women						
Wanted later	691	690	28.1	873	883	29.8
Not wanted at all	259	262	10.7	281	292	9.8
Desired waiting period till next birth (among sexually active women)						
< 12 months	61	52	2.1	116	112	3.8
12 – 23 months	66	50	2.0	109	88	3.0
24–47 months	549	528	21.5	611	577	19.5
48+ months	768	840	34.2	815	874	29.5
Infertile/want no more/others	1003	986	40.2	1295	1314	44.3

*N* Unweighted sample size, N* Weighted sample size, % Weighted sample proportion, ^a^ sexually active women are those who reported to had sex in the last 30 days at the time of the survey

**Fig. 1:**
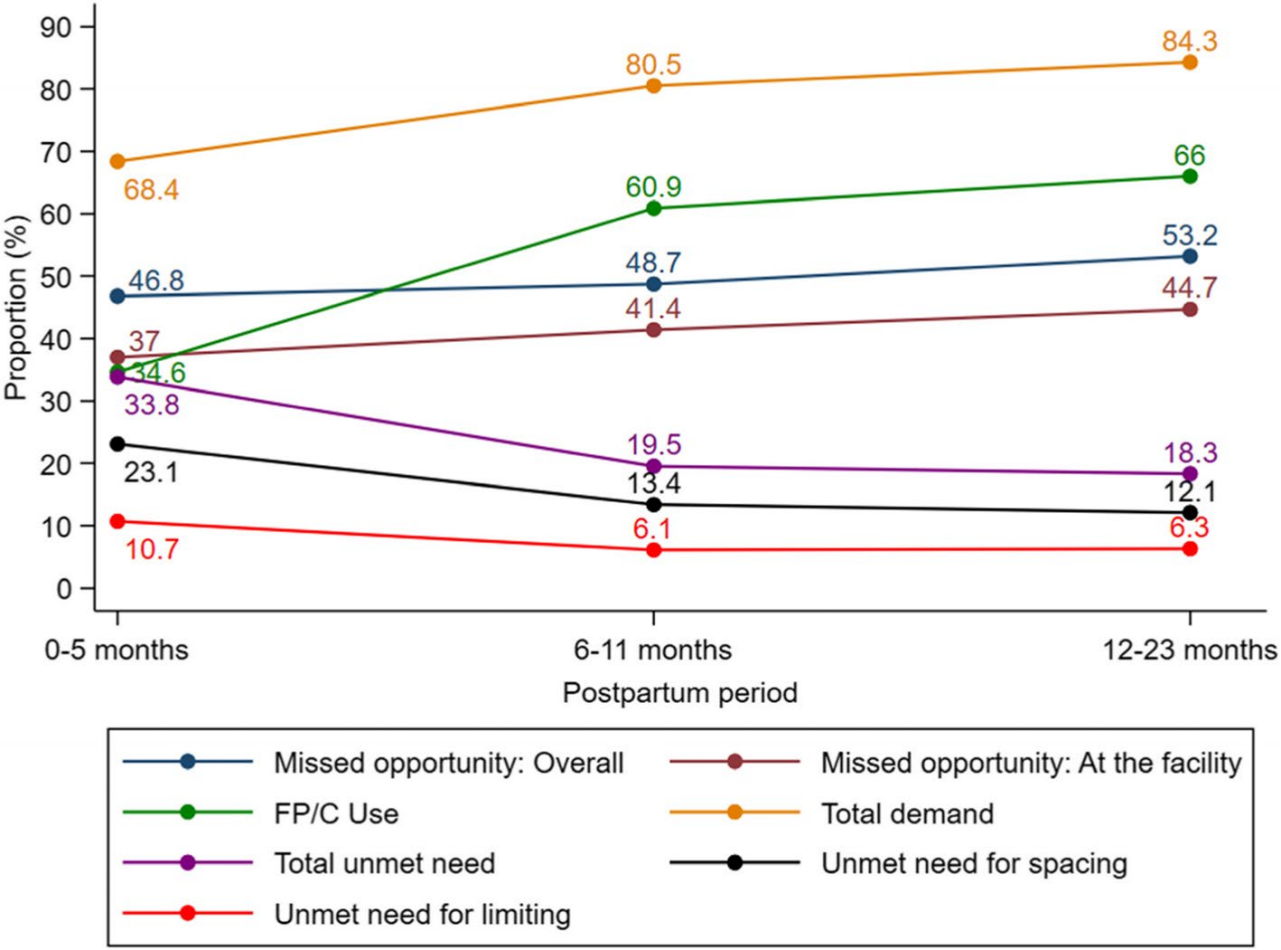
Missed opportunity, modern family planning/contraceptive (FP/C) use and Unmet need for family planning

Figure 1 and supplemental material Table 1 presents missed opportunity for FP/C counselling, contraceptive use and unmet need for family planning among women in the period 0 – 11 months and 12 – 23 months post-delivery. Missed opportunity for FP/C counselling increased with increasing duration post-delivery from 46.8% at 0-5 months to 53.2% at 12 – 23 months post-delivery (both at the facility and at the community level). The overall missed opportunity for FP/C counselling among women 12-23 months post-delivery (53.2%) was significantly higher than among women in the extended postpartum period (47.7%), *p* = 0.001. At the health facilities, about 37.0, 41.4 and 44.7% of the women 0 – 5 months, 6 – 11 months and 12 – 23 months post-delivery had missed opportunity for FP/C respectively. Missed opportunity for FP/C counselling at the health facilities among women 12-23 months post-delivery (44.7%) was significantly higher than among women in the extended postpartum period (39.2%), *p* = 0.003. Family planning/contraceptive use increases with increase in the duration post-delivery, while unmet need for FP/C decreases with increase in the duration post-delivery. Total demand for FP/C also increased with increase in the duration post-delivery, Fig. 1.

Table 3 shows bivariate analysis of current FP/C use and intendedness of last pregnancy among sexually active women. There is no significant association between the overall missed opportunity for FP/C counselling and modern contraceptive use among sexually active women in the extended postpartum period, *P* > 0.05. On the contrary, among women in the 12 – 23 months post-delivery period, there was a significant association between the overall missed opportunity for FP/C counselling and modern contraceptive use among sexually active women, *p* = 0.007. Missed opportunity for FP/C counselling was not significantly associated with the last birth being unintended among women in the extended postpartum period (*p* = 0.753) and among women 12 – 23 months post-delivery (*p* = 0.179). Similarly, unmet need for FP/C counselling was not significantly associated with missed opportunity for FP/C counselling among women in the extended postpartum period (*p* = 0.411) and among women 12 – 23 months post-delivery (*p* = 0.120).

**Table 3:** Association between missed opportunity, FP/C use, intendedness of last pregnancy and unmet need

	0 - 11 months (extended postpartum) N = 3803			12 - 23 month since last birth ***N*** = 3746		
	n (%)	OR (95% CI)	***p***-value	n (%)	OR (95% CI)	***p***-value
Modern contraceptive use						
Non users	997 (48.7)	Ref.		760 (57.1)		
Users	829 (46.7)	0.92 (0.79, 1.08)	0.323	1233 (51.0)	0.78 (0.65, 0.94)	0.007
Last pregnancy intendedness						
Intended	1037 (48.0)	Ref.		1178 (54.4)	Ref.	
Unintended	789 (47.4)	0.97 (0.82, 1.15)	0.753	815 (51.5)	0.89 (0.75, 1.06)	0.179
Total unmet need						
No unmet need	1301 (46.9)	Ref.		1596 (52.6)	Ref.	
Unmet need	496 (48.8)	1.08 (0.90, 1.29)	0.411	383 (55.8)	1.14 (0.92,1.41)	0.120

*n* Weighted sample with missed opportunity in the subgroup, % Weighted sample proportion, *OR* Crude Odds Ratio, *CI* Confidence Interval

Results of the univariate and multivariable analysis of the correlates of the overall missed opportunity for FP/C counselling among women 0 – 11 and 12-23 months postpartum are shown in Table 4. Bivariate analysis showed that rural/urban residence, education, wealth, parity, round of data collection and county of residence were all significant predictors of overall missed opportunity for FP/C counselling among women in the extended postpartum period, *p* < 0.05. When compared to urban residents, rural residents were more likely to have a missed opportunity for FP/C counselling, *p* = 0.032. Those with some level of formal education were less likely to have a missed opportunity for FP/C counselling, when compared to the reference group of women with no education. Women in the higher wealth quintiles were less likely to have a missed opportunity for FP/C counselling when compared to the reference group of women from the lowest wealth quintile. Those with 3 – 4 past births were less likely to have a missed opportunity for FP/C counselling when compared to the reference group of women with one or two past births. Women from Kericho, Nandi and West Pokot counties were more likely to have a missed opportunity for FP/C counselling when compared to women from Bungoma county, Table 4.

**Table 4:** Bivariate and multivariate analysis of overall (facility and community health worker) missed FP/C opportunity among women in the 0-11 months and 12-23 months postpartum periods

Characteristic	0 - 11 months (extended postpartum) – N = 3803				12 - 23 month since last birth – N = 3746			
	Bivariate analysis		Multivariable analysis		Bivariate analysis		Multivariable analysis	
	Odds Ratio (95% CI)	***p***-value	Adjusted Odds Ratio (95% CI)	***p***-value*	Odds Ratio (95% CI)	***p***-value	Adjusted Odds Ratio (95% CI)	***p***-value*
**Age group**								
15 – 19	Ref.	0.345	Ref.	0.488	Ref.	0.414	Ref.	0.772
20 – 24	0.99 (0.77, 1.27)		1.10 (0.83, 1.46)		0.98 (0.72, 1.32)		1.04 (0.75, 1.44)	
25 – 29	0.82 (0.63, 1.06)		0.95 (0.69, 1.30)		0.87 (0.65, 1.17)		0.97 (0.68, 1.36)	
30 – 34	0.94 (0.71, 1.25)		1.13 (0.79, 1.62)		0.83 (0.6, 1.15)		0.93 (0.62, 1.39)	
35 – 39	0.79 (0.56, 1.13)		0.92 (0.60, 1.42)		0.93 (0.65, 1.32)		0.98 (0.62, 1.54)	
40 – 45	0.74 (0.45, 1.21)		0.79 (0.44, 1.40)		1.30 (0.78, 2.16)		1.34 (0.75, 2.37)	
45 – 49	0.79 (0.29, 2.19)		0.72 (0.26, 2.01)		0.83 (0.41, 1.67)		0.75 (0.35, 1.63)	
**Marital Status**								
Never Married	Ref.	0.150	Ref.	0.546	Ref.	**0.027**	Ref.	0.073
Married/In a union	0.81 (0.66, 1.00)		0.90 (0.69, 1.17)		0.79 (0.63, 1.00)		0.77 (0.58, 1.03)	
Widowed/Divorced	0.87 (0.56, 1.37)		1.06 (0.65, 1.73)		1.12 (0.76, 1.66)		1.09 (0.72, 1.66)	
**Residence**								
Urban	Ref.	**0.032**	Ref.	0.327	Ref.	0.230	Ref.	0.411
Rural	1.27 (1.02, 1.57)*		1.14 (0.88, 1.48)		0.87 (0.70, 1.09)		0.9 (0.71, 1.15)	
**Education**								
No Education	Ref.	**0.016**	Ref.	0.086	Ref.	0.052	Ref.	0.138
Primary	0.52 (0.35, 0.78)**		0.63 (0.43, 0.92)*		0.60 (0.41, 0.88)*		0.74 (0.5, 1.1)	
Technical/vocational	0.42 (0.22, 0.81)*		0.56 (0.29, 1.09)		0.52 (0.27, 1.02)		0.64 (0.32, 1.29)	
Secondary	0.50 (0.33, 0.76)**		0.57 (0.38, 0.86)**		0.61 (0.40, 0.93)*		0.69 (0.44, 1.07)	
Higher	0.46 (0.29, 0.72)**		0.51 (0.31, 0.83)**		0.50 (0.32, 0.79)**		0.56 (0.35, 0.88)*	
**Wealth Quintile**								
Lowest	Ref.	**0.005**	Ref.	**0.023**	Ref.	0.845	Ref.	0.962
Lower	0.65 (0.52, 0.82)**		0.72 (0.57, 0.91)**		0.9 (0.71, 1.14)		1.01 (0.8, 1.28)	
Middle	0.69 (0.53, 0.89)**		0.88 (0.67, 1.15)		0.87 (0.68, 1.12)		0.96 (0.74, 1.26)	
Higher	0.70 (0.53, 0.91)**		0.98 (0.71, 1.35)		0.95 (0.72, 1.25)		0.91 (0.67, 1.25)	
Highest	0.67 (0.49, 0.92)*		1.10 (0.72, 1.67)		0.91 (0.64, 1.29)		0.89 (0.58, 1.36)	
**Parity**								
1 – 2	Ref.	**0.014**	Ref.	**0.026**	Ref.	0.121	Ref.	0.410
3 – 4	0.76 (0.64, 0.92)**		0.73 (0.58, 0.92)**		0.85 (0.71, 1.01)		0.87 (0.7, 1.09)	
5+	0.99 (0.78, 1.24)		0.86 (0.62, 1.19)		1.02 (0.83, 1.25)		0.96 (0.71, 1.29)	
**Sexually active**								
No	Ref.	0.501	Ref.	0.866	Ref.	0.205	Ref.	0.515
Yes	0.95 (0.81, 1.11)		1.02 (0.85, 1.21)		0.88 (0.73, 1.07)		1.08 (0.85, 1.37)	
**Data collection round**								
Jun/Jul 2014	Ref.	**0.016**	Ref.	**0.003**	Ref.	**0.014**	Ref.	**0.017**
Nov/Dec 2014	0.71 (0.49, 1.03)		0.69 (0.47, 1.01)		0.59 (0.38, 0.92)*		0.6 (0.39, 0.93)*	
Jun/Jul 2015	1.00 (0.70, 1.42)		1.02 (0.71, 1.45)		0.55 (0.37, 0.82)**		0.54 (0.36, 0.81)**	
Nov/Dec 2015	0.61 (0.39, 0.94)*		0.60 (0.38, 0.93)*		0.45 (0.29, 0.7)**		0.45 (0.29, 0.71)**	
Nov/Dec 2016	0.83 (0.60, 1.16)		0.68 (0.48, 0.96)*		0.71 (0.48, 1.04)		0.69 (0.46, 1.02)	
Nov/Dec 2017	1.12 (0.80, 1.56)		0.95 (0.66, 1.36)		0.70 (0.48, 1.04)		0.66 (0.44, 0.99)*	
Nov/Dec 2018	0.68 (0.48, 0.96)*		0.58 (0.40, 0.82)**		0.60 (0.40, 0.89)*		0.56 (0.37, 0.85)**	
**County**								
Bungoma	Ref.	**< 0.001**	Ref.	**< 0.001**	Ref.	**< 0.001**	Ref.	**< 0.001**
Kakamega	1.35 (0.79, 2.32)		1.46 (0.83, 2.58)		1.35 (0.83, 2.18)		1.32 (0.81, 2.14)	
Kericho	1.5 (1.01, 2.22)*		1.52 (1.03, 2.26)*		1.72 (1.15, 2.57)**		1.75 (1.18, 2.62)**	
Kilifi	1.27 (0.81, 1.99)		1.17 (0.76, 1.80)		1.54 (1.00, 2.38)*		1.43 (0.92, 2.21)	
Kiambu	0.98 (0.63, 1.53)		0.98 (0.60, 1.59)		1.84 (1.10, 3.07)*		1.89 (1.14, 3.14)*	
Kitui	0.76 (0.49, 1.19)		0.77 (0.49, 1.21)		1.17 (0.75, 1.83)		1.17 (0.74, 1.87)	
Nairobi	0.87 (0.56, 1.35)		0.88 (0.53, 1.47)		1.42 (0.91, 2.23)		1.49 (0.91, 2.43)	
Nandi	1.74 (1.12, 2.72)*		1.86 (1.18, 2.91)**		1.43 (0.92, 2.22)		1.42 (0.91, 2.21)	
Nyamira	1.02 (0.65, 1.61)		1.06 (0.67, 1.66)		1.14 (0.75, 1.74)		1.16 (0.76, 1.77)	
Siaya	0.79 (0.52, 1.23)		0.79 (0.51, 1.23)		0.68 (0.45, 1.01)		0.66 (0.43, 1.01)	
West pokot	2.97 (1.71, 5.14)**		2.34 (1.30, 4.22)**		2.82 (1.36, 5.82)**		2.43 (1.16, 5.11)*	

*p-value* Unadjusted Wald test *p-*value, p-value* adjusted Wald test *p-*value, *CI* Confidence interval, * Significant at 5% level of significance (los), ** - Significant at 1% los, Ref. – Reference category

As in bivariate analysis, age, marital status and sexual activity remained insignificant predictors of missed opportunity for FP/C counselling in the adjusted analysis. After controlling for all variables statistically significant on bivariate analysis in Table 4, rural/urban residence and education were no longer significant predictors of missed opportunity for FP/C counselling. The likelihood of having a missed opportunity for FP/C counselling was almost two-times among those who had no education as compared to those who had higher education (AOR = 1.96, *p* = 0.006). Within the wealth quintile categories, the only significant finding was that women from the lower quintile were less likely (AOR = 0.72, *p* = 0.005) to have a missed opportunity for FP/C counselling when compared to women in the lowest wealth quintile. Women with three to four past births were less likely to have a missed opportunity for FP/C counselling (AOR: 0.73, 95% CI: 0.58, 0.92, *p* = 0.009) when compared to the reference group of women with one or two past births. By county of residence, women from Kericho, Nandi and West Pokot counties were more likely to have a missed opportunity for FP/C counselling when compared to women from Bungoma county, *p* < 0.05. Round of data collection and county of residence were the only significant predictors of overall missed opportunity for FP/C counselling among women in the period 12 – 23 months post-delivery, *p* < 0.05.

Bivariate and multivariable analysis results for missed opportunity for FP/C at the facility are shown in Table 5. Parity, round of data collection and county of residence were the significant covariates of missed opportunity for FP/C counselling at the facility among women in the extended postpartum period. Women who had three to four previous births (adjusted OR: 0.71, *p* = 0.009) were less likely to have a missed opportunity for FP/C counselling at the facility when compared to women with one or two past births. By county of residence, women from Kericho, Nandi and West Pokot counties were 2-3 times more likely to have a missed opportunity for FP/C counselling at the facility when compared to women from Bungoma county, *p* < 0.05. Round of data collection and county of residence were the only significant predictors of overall missed opportunity for FP/C counselling at the facility among women in the period 12 – 23 months post-delivery, *p* < 0.05. Women from Kericho, Kilifi, Nairobi, Nandi and West Pokot counties were more likely to have a missed opportunity for FP/C counselling at the facility when compared to women from Bungoma county, *p* < 0.05.

**Table 5:** Bivariate and multivariate analysis of missed FP/C opportunity at the facility among women in the 0-11 months and 12-23 months postpartum periods

Characteristic	0 - 11 months (extended postpartum) – ***N*** = 3016				12 - 23 month since last birth – ***N*** = 2782			
	Bivariate analysis		Multivariable analysis		Bivariate analysis		Multivariable analysis	
	Odds Ratio (95% CI)	***p***-value	Adjusted Odds Ratio (95% CI)	***p***-value*	Odds Ratio (95% CI)	***p***-value	Adjusted Odds Ratio (95% CI)	***p***-value*
**Age group**								
15 – 19	Ref.	0.975	Ref.	0.791	Ref.	0.488	Ref.	0.313
20 – 24	1.07 (0.80, 1.42)		1.14 (0.83, 1.57)		1.10 (0.79, 1.55)		1.26 (0.88, 1.81)	
25 – 29	1.03 (0.76, 1.38)		1.17 (0.81, 1.67)		0.90 (0.64, 1.29)		1.03 (0.69, 1.53)	
30 – 34	1.05 (0.76, 1.46)		1.26 (0.84, 1.89)		0.91 (0.61, 1.34)		1.10 (0.68, 1.78)	
35 – 39	0.96 (0.66, 1.41)		1.05 (0.67, 1.67)		0.98 (0.65, 1.50)		1.22 (0.72, 2.07)	
40 – 45	0.83 (0.46, 1.51)		0.87 (0.43, 1.79)		1.21 (0.68, 2.17)		1.55 (0.82, 2.96)	
45 – 49	0.83 (0.25, 2.72)		0.68 (0.20, 2.26)		0.70 (0.30, 1.65)		0.71 (0.27, 1.89)	
**Marital Status**								
Never Married	Ref.	0.228	Ref.	0.656	Ref.	0.598	Ref.	0.641
Married/In a union	0.88 (0.69, 1.11)		0.97 (0.73, 1.30)		0.95 (0.72, 1.26)		0.95 (0.67, 1.35)	
Widowed/Divorced	0.63 (0.36, 1.09)		0.76 (0.41, 1.40)		1.15 (0.73, 1.81)		1.18 (0.71, 1.94)	
**Residence**								
Urban	Ref.	0.171	Ref.	0.930	Ref.	0.188	Ref.	0.676
Rural	1.19 (0.93, 1.52)		1.01 (0.75, 1.36)		0.84 (0.64, 1.09)		0.94 (0.71, 1.25)	
**Education**								
No Education	Ref.	0.344	Ref.	0.393	Ref.	0.863	Ref.	0.590
Primary	0.61 (0.37, 0.99)*		0.67 (0.43, 1.04)		0.99 (0.57, 1.73)		1.02 (0.59, 1.78)	
Technical/vocational	0.56 (0.26, 1.20)		0.61 (0.30, 1.22)		0.71 (0.31, 1.60)		0.60 (0.25, 1.42)	
Secondary	0.58 (0.35, 0.97)*		0.63 (0.39, 1.02)		0.96 (0.53, 1.73)		0.93 (0.51, 1.70)	
Higher	0.58 (0.33, 1.01)		0.57 (0.32, 1.00)*		0.92 (0.50, 1.70)		0.94 (0.51, 1.72)	
**Wealth Quintile**								
Lowest	Ref.	0.196	Ref.	0.139	Ref.	0.952	Ref.	0.998
Lower	0.74 (0.56, 0.98)*		0.81 (0.61, 1.07)		0.91 (0.69, 1.20)		0.96 (0.73, 1.27)	
Middle	0.71 (0.53, 0.96)*		0.90 (0.66, 1.22)		0.95 (0.71, 1.28)		1.00 (0.75, 1.33)	
Higher	0.72 (0.54, 0.97)*		1.02 (0.71, 1.46)		1.01 (0.73, 1.40)		0.95 (0.68, 1.34)	
Highest	0.78 (0.54, 1.12)		1.29 (0.82, 2.03)		1.00 (0.67, 1.50)		0.97 (0.61, 1.55)	
**Parity**								
1 – 2	Ref.	**0.039**	Ref.	**0.019**	Ref.	0.194	Ref.	0.523
3 – 4	0.78 (0.63, 0.96)*		0.71 (0.55, 0.92)**		0.84 (0.70, 1.02)		0.87 (0.68, 1.12)	
5+	1.03 (0.81, 1.32)		0.92 (0.65, 1.30)		0.88 (0.68, 1.13)		0.85 (0.59, 1.21)	
**Sexually active**								
No	Ref.	0.762	Ref.	0.952	Ref.	0.588	Ref.	0.533
Yes	0.97 (0.80, 1.18)		1.01 (0.81, 1.25)		0.94 (0.75, 1.18)		1.10 (0.82, 1.46)	
**Data collection round**								
Jun/Jul 2014	Ref.	**0.021**	Ref.	**0.004**	Ref.	**0.009**	Ref.	**0.011**
Nov/Dec 2014	0.73 (0.47, 1.12)		0.70 (0.46, 1.07)		0.77 (0.47, 1.27)		0.77 (0.47, 1.27)	
Jun/Jul 2015	1.00 (0.64, 1.58)		1.03 (0.67, 1.60)		0.55 (0.35, 0.87)		0.54 (0.35, 0.85)**	
Nov/Dec 2015	0.62 (0.38, 1.01)		0.61 (0.39, 0.97)*		0.46 (0.28, 0.77)		0.45 (0.27, 0.75)**	
Nov/Dec 2016	0.62 (0.41, 0.96)*		0.56 (0.36, 0.87)*		0.56 (0.37, 0.87)		0.61 (0.39, 0.96)*	
Nov/Dec 2017	0.77 (0.50, 1.18)		0.72 (0.47, 1.11)		0.51 (0.33, 0.78)		0.50 (0.32, 0.78)**	
Nov/Dec 2018	0.53 (0.35, 0.80)**		0.49 (0.33, 0.74)**		0.45 (0.29, 0.71)		0.47 (0.29, 0.76)**	
**County**								
Bungoma	Ref.	**< 0.001**	Ref.	**< 0.001**	Ref.	**0.006**	Ref.	**0.011**
Kakamega	1.15 (0.66, 2.00)		1.38 (0.78, 2.46)		0.76 (0.38, 1.52)		0.85 (0.42, 1.7)	
Kericho	2.12 (1.30, 3.45)**		2.04 (1.25, 3.33)**		2.45 (1.54, 3.90)		2.50 (1.56, 4.01)**	
Kilifi	1.76 (1.03, 3.00)*		1.55 (0.94, 2.56)		1.79 (1.09, 2.95)		1.79 (1.08, 2.98)*	
Kiambu	1.13 (0.66, 1.95)		0.96 (0.54, 1.71)		1.72 (0.91, 3.25)		1.60 (0.85, 3.00)	
Kitui	0.84 (0.50, 1.42)		0.82 (0.48, 1.40)		1.33 (0.78, 2.27)		1.40 (0.80, 2.47)	
Nairobi	1.14 (0.68, 1.90)		0.89 (0.50, 1.58)		1.93 (1.16, 3.20)		1.80 (1.04, 3.13)*	
Nandi	2.81 (1.70, 4.62)**		2.97 (1.79, 4.94)**		2.00 (1.22, 3.26)		2.07 (1.25, 3.43)**	
Nyamira	1.29 (0.75, 2.21)		1.34 (0.78, 2.29)		1.34 (0.81, 2.20)		1.33 (0.80, 2.21)	
Siaya	1.45 (0.88, 2.38)		1.46 (0.88, 2.43)		1.46 (0.94, 2.24)		1.43 (0.91, 2.27)	
West Pokot	2.62 (1.24, 5.56)*		2.40 (1.12, 5.15)*		2.10 (0.92, 4.77)		2.42 (1.04, 5.64)*	

*p-*value - Unadjusted Wald test *p-*value, *p-*value* - adjusted Wald test *p-*value, CI – Confidence interval, * - Significant at 5% level of significance (los), ** - Significant at 1% los, Ref. – Reference category

## Discussion

Our data show that among women in the extended postpartum period of 0-11 months, more than half were sexually active, had low FP/C use, had high unmet need, high demand for FP and most had experienced unintended pregnancies in the past. Though Kenya has made tremendous progress at the national level to increase mCPR from 39.4 in 2008/9 to 53.2% in 2014 [19] and to 60.8% in 2018 [27], there are missed opportunities for FP/C services which if addressed, could increase mCPR further. For women 0-23 months postdelivery who made contact with health care workers or were visited by community health workers (CHWs), those with missed opportunities for FP/C counselling increased from 47.7% for those 0-11 months to 53.2% for those 12-23 months. Other studies on postpartum FP have documented similar missed opportunities [18, 24]. The World Health Organization [15] and Gaffield et al. [14] recommends programs not to miss any opportunities to encourage postpartum FP across the continuum of care.

Use of FP during the extended-postpartum period in Kenya has increased from 41% in 2008 [28] to the current level of 50% while all method CPR has increased from 46 to 58% [19]. Our finding of increase in FP use between 0 and 5 months and 6-11 months has been documented previously [18, 29]. Using family planning calendar data from the Kenya Demographic health survey (DHS) of 2008/9, Winfrey and Kshitiz [29] showed a gradual increase in the use of postpartum FP from 8.7% in month one to 35.8% in month 12 postpartum. Using DHS data, Hounton et al. 2015 [30] found increases in use of FP 3-months postpartum in Ethiopia from 5 to 12%, Malawi from 9.5 to 14.2% and a decrease in Nigeria from 5.9 to 3.8% from 2008 to 2013. Though not comparable to Kenya since general CPR is considerably higher than in Ethiopia, Malawi and Nigeria, the reported increase shows potential to increase postpartum FP use while the decrease in Nigeria is a reminder that countries must remain focused on maintaining attained gains.

At the time of the survey, less than half of the women 0-11 months postpartum (46.5%) and 64.5% of women 12 – 23 months postpartum were using modern contraception methods. There is high level of unmet need for FP/C among women 0-11 months postpartum (26.8%) and among women 12 – 23 months postpartum (18.4%) which are higher than the national average of 17.5% in 2014 [19, 24]. Similar high levels of unmet need have been reported in Kenya for limiting at 36 and 48% for birth spacing among postpartum women [31]. Overall, the unmet need for FP during the extended postpartum period has decreased from 75.2% in 1998 [32] to 26.8% in 2018 as documented in this study. There is still room to improve provision of postpartum FP in Kenya. Some of the suggested solutions involve developing strategies to address the inequalities caused by socio-economic factors and the integration of FP with maternal and new born health services, particularly with childbirth in facilities and child immunization [23, 33–35]. Dulli et al. [33] cluster randomized controlled trial in Rwanda showed that integration of FP into immunization services was successful in increasing mCPR without negatively impacting immunization rates. Hamon et al. [34] found that the integration of FP into routine outreach services could improve acceptability and availability of FP services. The MOMI project demonstrated the potential to shift demand for postpartum care services in the postpartum period by using CHWs [35].

Though interventions to address missed opportunities to reduce unmet contraceptive need would seem straightforward, programs have to navigate potential barriers. Duysburg et al. [17] noted that availability of commodities or services does not translate to postpartum FP use among postpartum women. Ochako et al. [36] found that awareness and knowledge about contraception does not necessarily translate to use among women aged 15-24 years, pointing to potential influence of social networks and other barriers. Djellouli et al. [35] also noted the complex interplay between social capital (the relationships between people within the community) and the development of bonding social capital to become a community norm allowing women to seek postpartum care services. A multipronged approach will be necessary if FP programs are going to capitalize on missed opportunities to reduce unmet contraceptive need. Among the many options to consider will be tapping into the potential of CHWs to be agents of social change and promote postpartum FP use [35] and integration of postpartum FP into other services where capacity exists to ensure quality of primary services are not compromised [33].

There are some limitations which need to be considered when interpreting the data. By use of cross-sectional design, we were limited in our ability to examine whether reported missed opportunities resulted in unintended pregnancies. There is a risk of recall bias, particularly for women who delivered at the beginning of the 2-year reference period. Since only individual-level factors were examined in this study, it is possible that post-partum FP practice is influenced by community-level factors that were not measured. For example, community norms about contraceptive use may positively or negatively influence an individual’s contraceptive behaviours. Additionally, being a household survey, there are no facility data which would have allowed determination of integration of FP and reproductive health services. There are strengths to this survey which include inclusion of a large sample of women, use of data collectors without medical training to reduce bias and real-time data collection with sufficient controls for quality assurance. In addition, pooled data from seven-rounds of survey were included. Necessary statistical adjustment including svyset command in Stata designating the round number as a stratum were used to take care of clustering by round since the women included were not independently sampled.

## Conclusion

In conclusion, given the vulnerabilities of women during the extended postpartum period, programs specifically addressing the needs of postpartum women need to be strengthened. Though Kenya is making progress meeting the needs of women who desire to use family planning, there is need for implementing innovative interventions to address missed opportunities during the extended postpartum period. Strengthening health systems, integrating service delivery for the postpartum period and promoting demand for postpartum contraception (PPC) through community interventions offer potential for success. Similarly, the findings regarding high rates of unmet need, which are consistent across different locations in Kenya, point to the need for further research for interventions to improve access to family planning services. Using a longitudinal study will be useful to understand the context of the contacts as well as the quality the women have with HCW and CHW to maximize use of these opportunities to increase mCPR.

## Supplementary Information

**Additional file 1: Supplemental materials. Table 1.** Missed opportunities for FP/C counselling, FP use and unmet need for FP. **Figure 1.** Pictorial representation of missed opportunity definition (All participants). **Figure 2.** Pictorial representation of missed opportunity definition (Women in the extended postpartum period). **Figure 3.** Pictorial representation of missed opportunity definition (Women 12-23 months post-delivery).

## Data Availability

The datasets generated during the study are publicly available from the PMA website (https://www.pma2020.org/request-access-to-datasets).

## Abbreviations

AOR: Adjusted Odds Ratio
CHW: Community Health workers
DHS: Demographic and Health Survey
FP/C: Family Planning/Contraception
HCW: Health Care Workers
mCPR: Modern Contraceptive Prevalence Rate
PMA2020: Performance Monitoring and Accountability 2020
PPC: Postpartum Care
