# Supplementary material for: Missed opportunities for family planning counselling among postpartum women in eleven counties in Kenya
Source: BMC Public Health. 2022 Feb 8;22:253. doi: 10.1186/s12889-022-12623-0 (PMC8822701; doi:10.1186/s12889-022-12623-0)
Supplement: Supplementary file 1 — Additional file 1: Supplemental materials. Table 1. Missed opportunities for FP/C counselling, FP use and unmet need for FP. Figure 1. Pictorial representation of missed opportunity definition (All participants). Figure 2. Pictorial representation of missed opportunity definition (Women in the extended postpartum period). Figure 3. Pictorial representation of missed opportunity definition (Women 12-23 months post-delivery). [file 12889_2022_12623_MOESM1_ESM.docx]

**Missed Opportunities for Family Planning counselling among Postpartum Women in eleven counties in Kenya**

Mary N. Thiongo^1*^, Peter B. Gichangi^2*^, Michael Waithaka^1^, Amy Tsui^3^, Linnea A. Zimmerman^3^, Scott Radloff^3^, Marleen Temmerman^4^, Saifuddin Ahmed^3^

1. International Centre for Reproductive Health, Kenya.
2. Technical University of Mombasa, Mombasa, Kenya.
3. Department of Population, Family and Reproductive Health, Johns Hopkins Bloomberg school of public Health, Baltimore, MD, USA.
4. Aga Khan University, Kenya.

*These authors have equal contribution

Corresponding author: Prof Peter Gichangi, P.O. Box 2631-00202, Nairobi, Kenya; email: gichangip2015@gmail.com; +25472521946

**Supplemental materials**

**Table 1: Missed opportunities for FP/C counselling, FP use and unmet need for FP**

|  | **Postpartum Period (months)** | | | | | |
| --- | --- | --- | --- | --- | --- | --- |
|  | **0 – 11** | | **12 - 23** | | **Total 0 - 23** | |
|  | **N*** | **%** | **N*** | **%** | **N*** | **%** |
| Number of women | 3,825 |  | 3,746 |  | 7,571 |  |
| Overall Missed opportunity for FP | 1,826 | 47.7 | 1,992 | 53.2 | 3,818 | 50.4 |
| Missed opportunity for FP at the Facility^£^ | 1,190 | 39.2 | 1,245 | 44.7 | 2,435 | 41.8 |
| CP use | 1,836 | 48.0 | 2,473 | 66.0 | 4,308 | 56.9 |
| MCP | 1,777 | 46.5 | 2,417 | 64.5 | 4,194 | 55.4 |
| TCP | 61 | 1.6 | 56 | 1.5 | 117 | 1.5 |
| Total Unmet need | 1,016 | 26.8 | 686 | 18.4 | 1,702 | 22.7 |
| Unmet need for spacing | 696 | 18.2 | 451 | 12.1 | 1,148 | 15.2 |
| Unmet need for limiting | 319 | 8.3 | 234 | 6.3 | 554 | 7.3 |

N* – Weighted sample size, % – Weighted sample proportion, ^£^ - Calculated out of the total number of women who visited a health facility, CP – Contraceptive use, FP – Family Planning, MCP – Modern Contraceptive Prevalence, TCP – Traditional Contraceptive Prevalence

**Figure 1: Pictorial representation of missed opportunity definition (All participants)**

Definition 1: Overall missed opportunity = $\frac{(B + C)}{\begin{aligned} The total number of postpartum \\ women \end{aligned}}$

Definition 2: Missed opportunity at the facility = $\frac{A}{\begin{aligned} Postpartum women who visited a \\ facility in the last 12 months \end{aligned}}$

**Figure 2: Pictorial representation of missed opportunity definition (Women in the extended postpartum period)**

Definition 1: Overall missed opportunity = $\frac{(B + C)}{\begin{aligned} The total number of postpartum \\ women \end{aligned}}$

Definition 2: Missed opportunity at the facility = $\frac{A}{\begin{aligned} Postpartum women who visited a \\ facility in the last 12 months \end{aligned}}$

**Figure 3: Pictorial representation of missed opportunity definition (Women 12-23 months post-delivery)**

Definition 1: Overall missed opportunity = $\frac{(B + C)}{\begin{aligned} The total number of postpartum \\ women \end{aligned}}$

Definition 2: Missed opportunity at the facility = $\frac{A}{\begin{aligned} Postpartum women who visited a \\ facility in the last 12 months \end{aligned}}$
